# Prognostic Impact of Tricuspid Regurgitation in Patients Undergoing Aortic Valve Surgery for Aortic Stenosis

**DOI:** 10.1371/journal.pone.0136024

**Published:** 2015-08-20

**Authors:** Julia Mascherbauer, Andreas A. Kammerlander, Beatrice A. Marzluf, Alexandra Graf, Alfred Kocher, Diana Bonderman

**Affiliations:** 1 Department of Cardiology, Medical University of Vienna, Vienna, Austria; 2 Department of Thoracic Surgery, Otto Wagner Hospital, Vienna, Austria; 3 Department of Medical Statistics, Medical University of Vienna, Vienna, Austria; 4 Department of Cardiac Surgery, Medical University of Vienna, Vienna, Austria; University of Louisville, UNITED STATES

## Abstract

**Background:**

The prognostic significance of tricuspid regurgitation (TR) and right ventricular (RV) function in patients undergoing aortic valve replacement (AVR) for severe aortic stenosis (AS) is unknown. The aim of the present study was to evaluate the impact of TR and RV systolic dysfunction on early and late mortality in this setting.

**Methods:**

This was a prospective single-center observational study. 465 consecutive patients who were referred to AVR for severe AS were investigated. Significant TR was defined as TR≥moderate by transthoracic echocardiography.

**Results:**

At baseline, significant TR was present in 26 (5.6%) patients. Patients with TR presented with a higher EuroSCORE I (p = 0.001), a higher incidence of previous cardiac surgery (p<0.001), pulmonary hypertension (p = 0.003), more dilated RVs (p = 0.001), and more frequent RV dysfunction (p = 0.001). Patients were followed for an average of 5.2 (±2.8 SD) years. By multivariable Cox regression analysis TR (p = 0.014), RV dysfunction (p = 0.046), age (p = 0.001) and concomitant coronary artery bypass graft surgery (CABG, p = 0.003) were independently associated with overall mortality. By Kaplan-Meier analysis, survival rates were significantly worse in patients with significant than with non-significant TR (log rank p = 0.001).

**Conclusions:**

TR, RV dysfunction, age, and concomitant CABG are associated with outcome in patients undergoing AVR for severe AS. This finding underlines the importance of a thorough echocardiographic evaluation with particular consideration of the right heart in these patients.

## Introduction

The prognostic impact of tricuspid regurgitation (TR) in patients undergoing aortic valve replacement (AVR) for isolated severe aortic stenosis (AS) is unknown. The few existing studies on the prognostic significance of TR in various other disease settings, however, indicate a strong association of TR with clinical outcome [1]. TR is associated with a poor prognosis after mitral valve interventions like percutaneous balloon valvuloplasty [2] or mitral valve repair and replacement [3, 4]. A retrospective study of a large echocardiographic data file reported a significantly increased mortality among patients with moderate and severe TR, which was independent of left ventricular ejection fraction or pulmonary artery pressure [5]. TR has also been shown to indicate a dismal prognosis in patients with flail tricuspid leaflet due to trauma [6], in heart failure patients [7–9], and in patients late after left heart valve surgery [10]. However, our own work showed no independent association of TR with mortality by multivariable analysis [9, 10], while right ventricular (RV) systolic dysfunction was associated with outcome in patients, who previously underwent left heart valve surgery [10].

The present study evaluated the predictive value of TR and/or RV systolic dysfunction prior to AVR in 465 AS patients.

## Methods

### Study population

Between January 1998 and January 2005 465 consecutive patients with isolated severe AS necessitating valve replacement who agreed to participate were included in this observational, non-interventional study.

Patients who needed emergent surgery and those with moderate and severe mitral regurgitation were excluded. Furthermore, patients who were primarily referred to coronary artery bypass graft (CABG) surgery and additionally received an aortic prosthesis for AS were also excluded.

Clinical, echocardiographic, operative and outcome data were prospectively collected in a computerized database.

### Ethics statement

The ethics committee of the Medical University of Vienna approved the present study “Ergebnisse nach Herzklappenoperationen” (engl. “Results after Heart Valve Surgery”), including the consent procedure. Verbal informed consent was obtained from the patients for participation in the study and was documented in the medical records.

### Patient evaluation and follow up

All patients suffered from severe AS (peak velocity ≥4 m/s, mean pressure gradient ≥40 mmHg, calculated valve area <1.0 cm^2^ in the presence of normal left ventricular function) and presented with symptoms (exertional dyspnea ≥ functional NYHA class II, exertional angina pectoris ≥ functional CCS class II, syncope) or with reduced left ventricular function (ejection fraction ≤50%).

Patients were re-evaluated three to nine months after AVR, and at intervals between one and three years thereafter.

Short-term mortality was defined as death from any cause within 30 days after operation, if the patient was discharged from hospital or within any interval, if the patient was not discharged. Any death occurring at least 30 days after surgery was defined as late mortality.

For the assessment of outcome, the primary endpoint was death. Short-term as well as late deaths were included in the analysis. Deaths were classified as cardiac or non-cardiac on the basis of review of medical records, including autopsy records and death certificates, which were available in all cases.

### Echocardiography

All patients underwent a comprehensive echocardiographic examination including M-mode echocardiography, 2-dimensional echocardiography, and conventional and color Doppler ultrasonography.

TR was quantified by an integrated approach (Table 1) [11, 12]. Moderate and severe TR were considered significant and were compared with trace and mild TR. The graduation into non-significant TR (trace/mild) and significant TR (moderate/severe) was chosen to account for inaccuracies due to the semiquantitative assessment of TR by echocardiography and has previously been deemed reasonable [1, 3, 5, 9].

**Table 1:** Echocardiographic and Doppler parameters used for grading of TR severity.

Parameter	Mild	Moderate	Severe
Tricuspid valve	Normal	Normal or abnormal	Abnormal / flail leaflet / poor coaptation
RV/RA/IVC size	Normal*	Normal or dilated	Dilated
VC width [mm]	Not defined	Not defined, but ≤7	>7
PISA radius [mm]	≤5	6–9	>9
Hepatic vein flow	Systolic dominance	Systolic blunting	Systolic reversal

Presence of three or more of the five parameters shown defines severity grade (mild, moderate or severe).

RV indicates right ventricular; RA, right atrium; IVC, inferior vena cava; VC, vena contracta; PISA, proximal isovelocity surface area.

* Unless there are other reasons for RV or RA enlargement (e.g. pulmonary hypertension, pulmonary valve disease).

Based on current guideline recommendations for the diagnosis and treatment of pulmonary hypertension (PH) [13], PH was considered likely, if a peak TR velocity >3.4m/s (estimated systolic pulmonary artery pressure >50mmH) was present.

RV function was assessed according to recent recommendations by including information on size, visual assessment of contractility, and tricuspid annular plane systolic excursion (TAPSE) [14]. RV dysfunction was defined as TAPSE <16mm.

### Statistical analysis

Statistical analysis was performed using SAS 9.1 for Windows (SAS statistical software, SAS Institute, Cary, NC, USA). Continuous variables were summarized as mean ± standard deviation, median and quartiles; categorical variables were summarized as percentages. Differences in baseline characteristics, echocardiographic, and operative data between patients with or without significant TR were assessed using a t-test for continuous data and a chi-squared test for categorical data.

Kaplan-Meier estimates were used to calculate 1, 3, 5, and 7-year survival rates. Differences in these and all other tests were considered significant at p <0.05.

To determine influence factors on overall mortality, univariate Cox regression models for each influence variable were performed. Additionally, multiple stepwise Cox regression analyses were performed accounting for all variables being significant (p<0.05) in the univariate analyses. Similar analysis was performed for operative mortality, using logistic regression models instead of Cox regression models.

The following influence factors were assessed: Age, body surface area, enddiastolic diameter of left and RV (continuous variables); NYHA and CCS functional class, additive EuroSCORE I (ordinal variables); gender, PH, significant TR, renal failure, concomitant CABG, extracardiac arteriopathy, chronic obstructive pulmonary disease, diabetes mellitus, coronary artery disease, left ventricular ejection fraction ≤50%, previous cardiac surgery and RV dysfunction (factor variables).

To validate the influence factors found by the statistical analysis described above, we additionally performed variable selection for the final model using bootstrap methods. For each of 5000 bootstrap samples we calculated univariable and corresponding multivariable regression models with stepwise selection (as for the overall sample). The final model was then performed with the influence factors being selected with the multivariable model in more than 60% of the bootstrap samples.

## Results

### Clinical characteristics at baseline

Table 2 shows the baseline characteristics of the 465 patients. Significant TR was present in 26 (5.6%) patients, and was interpreted as being secondary to the left heart disease in all cases. 11 patients presented with mildly or moderately reduced RV function by echocardiography at baseline.

**Table 2:** Baseline patient characteristics

	All patients	Non-significant TR	Significant TR	p
	n = 465	n = 439 (94%)	n = 26 (6%)	
Age [years]	69.8±10.0	69.6 ± 9.9	72.0 ± 10.4	0.233
Female [n/(%)]	246 (53)	227 (52)	19 (73)	0.034
BSA [m^2^]	1.87±0.23	1.88 ± 0.23	1.80 ± 0.24	0.117
Additive EuroSCORE I [%]	6.25±2.50	6.15 ± 2.47	7.81 ± 2.48	0.001
Previous cardiac surgery [n/(%)]	32 (7)	25 (6)	7 (27)	0.001
Previous valve surgery [n/(%)]	14 (3)	11 (3)	3 (12)	0.003
CAD [n / (%)]	176 (38)	165 (38)	11 (42)	0.642
CAD ≥2 vessels [n/(%)]	45 (10)	42 (10)	3 (12)	0.701
Diabetes [n/(%)]	68 (15)	62 (14)	6 (23)	0.075
COPD [n/(%)]	45 (10)	44 (10)	1 (4)	0.299
Serum creatinine [mmol/l]	1.11±0.68	1.09±0.67	1.4±0.89	0.328
Renal failure [n/(%)]	12 (3)	11 (3)	1 (4)	0.647
Extracard. artheriopathy [n/(%)]	70 (15)	66 (15)	4 (15)	0.969
Pacemaker [n/(%)]	12 (3)	9 (2)	3 (12)	0.002
NYHA [functional class]	2.34±0.68	2.32±0.68	2.54±0.56	0.117
NYHA ≥III [n/(%)]	108 (23)	103 (24)	5 (19)	0.951
**Echocardiographic data**				
Peak AV velocity [m/s]	5.00±0.77	5.02±0.77	4.49±0.82	0.007
Mean AV gradient [mmHg]	65.90±19.33	66.46±19.36	56.19±16.34	0.011
AV area [cm^2^]	0.62±0.17	0.62±0.17	0.58±0.10	0.350
LVEF <50% [n/(%)]	53 (11)	48 (11)	5 (19)	0.196
RV dysfunction [n/(%)]	11 (2)	1 (0.2)	10 (38)	0.001
LV_EDD_ [mm]	47.90±7.07	47.91±7.10	47.79±6.62	0.938
RV_EDD_ [mm]	31.17±4.72	30.99±4.58	34.33±5.94	0.001
LA [mm]	56.32±7.99	56.00±7.72	61.44±10.52	0.001
RA [mm]	53.74±20.52	53.25±20.85	61.72±11.71	0.002
IVS [mm]	16.19±2.67	16.27±2.67	14.60±2.20	0.018
sPAP >50mmHg [n/(%)]	39 (8)	32 (7)	7 (27)	0.001

Values are expressed as mean±SD or frequency (%). BSA indicates body surface area; BMI, body mass index; CAD, coronary artery disease, defined as any degree of coronary lumen diameter narrowing; ≥2 vessels, affecting two or three coronary arteries; COPD, chronic obstructive pulmonary disease; NYHA, New York Heart Association; AV, aortic valve; LVEF, left ventricular ejection fraction; LV, left ventricle; RV, right ventricle; EDD, end-diastolic diameter from the apical 4-chamber view; LA, left atrial longitudinal diameter from the apical 4-chamber view; RA, right atrial longitudinal diameter from the apical 4-chamber view; IVS, interventricular septal thickness by M-Mode from the parasternal short-axis view; sPAP, systolic pulmonary artery pressure.

Patients with significant TR were more often female (p = 0.034), presented with a higher additive EuroSCORE (p = 0.001), a higher incidence of previous cardiac surgery (p<0.001), and previous valve surgery (p = 0.003), and a higher frequency of PH (p = 0.003). Only 12 patients in the cohort carried implanted pacemakers prior to surgery, three of them among patients with significant TR.

### Echocardiographic data at baseline

Baseline echocardiographic data are shown in Table 2. 19% of patients with significant TR were found to have left ventricular ejection fractions <50%, compared with 11% in patients without significant TR (p = 0.196). Patients with significant TR presented with significantly larger RV (p = 0.001), worse RV function (p = 0.001), and larger left (p = 0.001) and right atria (p = 0.002). Systolic pulmonary artery pressures were higher in patients with significant TR (p = 0.002), and PH was more frequent (27% versus 7%, p<0.001).

Left atrial size, reflecting left ventricular filling pressure, was significantly correlated with peak TR velocity, (R = 0.381, p<0.0001).

### Operative data

Operative data are displayed in Table 3. There was no difference between patients with and without significant TR with respect to additional CABG, use of a mechanical prosthesis, aortic root enlargement or replacement. Three patients (12%) of those with significant TR underwent tricuspid valve repair at the time of AVR (p = 0.257).

**Table 3:** Operative data.

	All patients	Non-significant TR	Significant TR	p
	n = 465	n = 439 (94%)	n = 26 (6%)	
CABG [n/(%)]	113 (24)	107 (24)	6 (23)	0.861
CABG ≥2 grafts [n/(%)]	38 (8)	35 (8)	3 (12)	0.327
Mechanical prosthesis [n/(%)]	112 (24)	106 (24)	6 (23)	0.901
Aortic root enlargement [n/(%)]	13 (3)	13 (3)	0	0.374
Aortic root replacement [n/(%)]	17 (4)	16 (4)	1 (4)	0.967
Additional TV repair [n/(%)]	3 (0.7)	0	3 (12)	0.257

Values are expressed as numbers or frequency (%). CABG indicates coronary artery bypass graft; TV, tricuspid valve.

### Short-term mortality

28 short-term deaths (6.0%) were observed. Causes of deaths are shown in Table 4. Short-term mortality was significantly higher among patients with significant TR (19% versus 5%, p = 0.004).

**Table 4:** Causes of death.

Mortality	All patients	Non-significant TR	Significant TR	p
	n = 465	n = 439 (94%)	n = 26 (6%)	
**Short-term**				
Cardiac	23	19	4	
Infection	3	3		
Hemorrhage	2	1	1	
	28 (6%)	23 (5%)	5 (19%)	0.004
**Late**				
Cardiac	32	30	2	
Cancer	21	19	2	
Infection	7	6	1	
Stroke	6	6		
Pulmonary embolism	3	3		
Lung failure	3	3		
Renal failure	2	1	1	
Liver failure	2	2		
Multiple Organ Failure	2	2		
Car accident	1	1		
Unknown	1	1		
	80 (17%)	74 (17%)	6 (23%)	0.414
**Overall Mortality**	108 (23%)	97 (22%)	11 (42%)	0.018

Values are expressed as numbers or frequency (%).

The variables found to be associated with short-term mortality in the univariable analysis were age (p = 0.005), body surface area (p = 0.007), EuroSCORE (p<0.001), significant TR (p = 0.007), concomitant CABG (p = 0.001), coronary artery disease (p = 0.002) and previous cardiac surgery (p<0.001). In the multivariable model, only EuroSCORE (p = 0.007), CABG (p = 0.002) and previous cardiac surgery (p = 0.009) remained significant predictors of short-term mortality. However, using variable selection by the bootstrap method, only the result for previous cardiac surgery (p<0.001) was approved.

#### Overall Outcome

Follow-up was complete in 99.8% of patients. Patients were followed for an average of 5.2 (SD ±2.8) years. 108 late deaths were observed. Late mortality was not significantly different between patients with and without significant TR pre-operatively (p = 0.414, Table 4).

**Fig 1:**
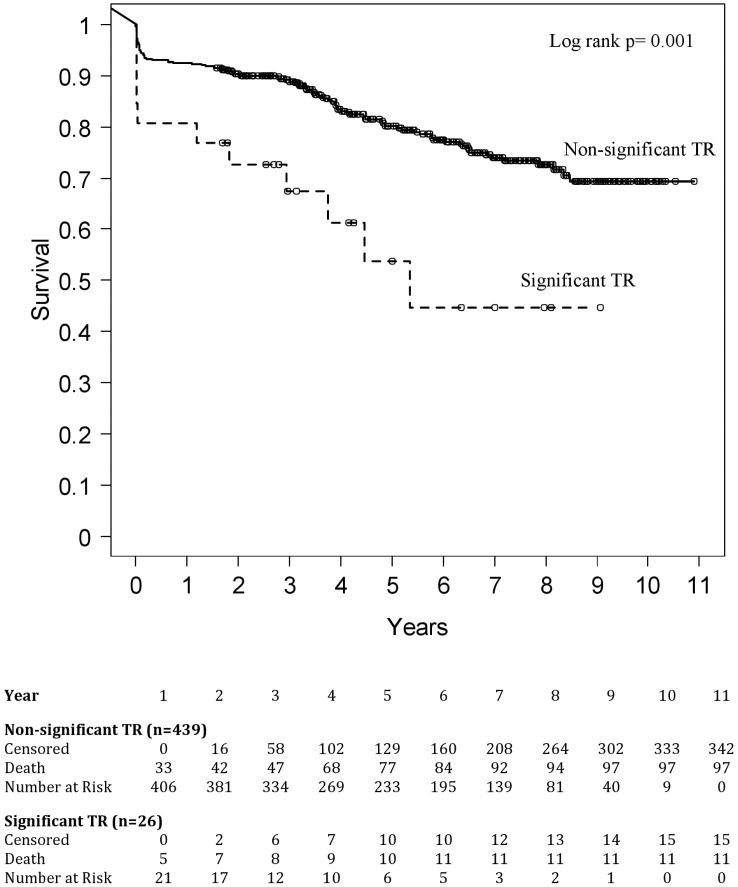
Overall survival in patients with versus without significant TR. TR indicates tricuspid regurgitation.

Overall survival was worse in patients with significant TR, driven by a higher short-term mortality (Fig 1). 1-, 3-, 5-, and 7-year survival rates were 81%, 68%, 54% and 45% versus 93%, 89%, 80%, and 74% in patients with no or mild TR (log rank p = 0.001).

Using univariate Cox regression analysis, overall outcome was determined by age (p<0.001), body surface area (p = 0.006), preoperative NYHA functional class (p = 0.048), EuroSCORE (p<0.001), PH (p = 0.009), significant TR (p = 0.001), RV dysfunction (p = 0.015), CABG (p = 0.001), diabetes (p = 0.010), coronary artery disease (p = 0.002), and previous cardiac surgery (p = 0.017).

By multiple Cox regression analysis, only age (p = 0.001), CABG (p = 0.003), significant TR (p = 0.014), and RV dysfunction (p = 0.046) remained significant predictors of overall mortality. The results of the multivariate analysis are shown in Table 5.

**Table 5:** Multivariate regression analysis of factors influencing overall mortality.

Parameter	Parameter Estimate	Standard Error	p-value	Hazard Ratio	95% Confidence Limits	
Age	0.041	0.012	0.001	1.042	1.017	1.068
CABG	0.650	0.216	0.003	1.916	1.256	2.925
Significant TR	0.910	0.372	0.014	2.484	1.199	5.145
RV dysfunction	1.731	0.868	0.046	5.647	1.030	30.959

CABG indicates coronary artery bypass graft; TR, tricuspid regurgitation; RV, right ventricle

Using bootstrap methods for variable selection approved the influence of significant TR (p = 0.007) and CABG (p = 0.006) on overall mortality, which were also identified by the conventional statistical analysis described above. Neither age, nor previous cardiac surgery or RV dysfunction were selected for the final model.

Symptoms and functional status at last follow-up were significantly worse in patients with significant pre-operative TR (1.68±0.75 versus 1.48±0.65, p = 0.03; data not shown).

## Discussion

We studied a cohort of 465 consecutive patients who underwent AVR for severe AS. Significant TR and RV dysfunction pre-operatively were both found to be independently associated with overall outcome. This novel observation provides important additional information for the risk assessment of the growing AS patient population.

Existing evidence suggests that TR is not a benign disorder and carries a considerable impact on morbidity and mortality [1]. TR has been shown to be associated with poorer outcome in heart failure patients [7–9], patients undergoing mitral balloon valvuloplasty [2], and mitral valve surgery [3, 4]. In addition, a large retrospective study reported a significantly increased mortality among patients with moderate and severe TR, independent of left ventricular ejection fraction or pulmonary artery pressure [5].

The present study is dedicated to the prognostic impact of significant pre-operative TR in patients undergoing AVR for isolated severe AS, which has not been assessed so far. The development of TR in this setting is the consequence of a pathogenetic process involving the left ventricle, the pulmonary vascular bed, and finally the RV. Due to the outflow obstruction, left ventricular filling pressures rise and cause a subsequent increase of left atrial and capillary wedge pressures. Pulmonary vascular compliance declines and adds to the increasing resistance against the RV, causing an increase in RV end-diastolic pressure. Two additional mechanisms may aggravate RV pressure overload: diffuse fibrosis / extracellular matrix expansion of the left ventricular myocardium (particularly in patients with AS [15, 16]), leading to a further increase in left atrial pressure, and remodeling of the pulmonary vascular bed.

The RV eventually fails to compensate the pressure overload and dilates. Tricuspid annular dilatation finally leads to the development of TR [10]. The occurrence of significant TR initiates a vicious circle propagating further RV dilatation and dysfunction, more tricuspid annular dilatation, and consequently worsening of TR [17]. An additional mechanism potentially contributing to TR evolution is the retraction of valve leaflets by pacemaker leads. In the present study significantly more patients with relevant TR were pacemaker carriers (Table 2).

RV dysfunction was independently associated with mortality in the Cox regression model. However, after bootstrap analysis, significant TR was the most robust parameter associated with adverse outcome. Our data suggest that TR is an indicator of RV uncoupling from its afterload consisting of left ventricular filling resistance, pulmonary vascular resistance and compliance. Bearing in mind this pathophysiologic cascade, TR stands at the end of a disease process, which in its course includes the progression of myocardial fibrosis and stiffness, both found to impact on mortality and morbidity in AS [15, 16].

It has previously been shown that PH in isolated AS is related to elevated left ventricular end-diastolic pressures [18]. Data on the impact of PH in patients undergoing AVR, however, are sparse. Several authors have pointed out that patients with AS and PH benefit from AVR compared with conservative treatment [19–23]. Only two recent papers explicitly addressed the impact of PH on outcome after AVR [24, 25]. Both studies are based on retrospective analyses and report controversial results. Zuern and coworkers [24] included only 176 patients who underwent AVR. Patients with systolic pulmonary artery pressures >30mmHg had a significantly increased 5-year mortality rate. Melby and coauthors [25] studied 1080 patients who underwent AVR and found a significant impact of PH on operative mortality and long-term survival. However, after exclusion of those 102 patients who had concomitant valve surgery in addition to AVR, operative mortality was not significantly different between patients with or without PH.

Our own data indicate no independent influence of PH as determined by echocardiography on short- or long-term mortality. TR and RV dysfunction on the contrary, were significantly associated with outcome in the multivariable analysis. One explanation for this observation might be the imprecision of Doppler echocardiography as described by Fisher et al [26]. Inaccurate Doppler estimates of pulmonary artery systolic pressure were reported in nearly 50% of cases, with similar frequency of over-and underestimation, leading to frequent misclassification of the severity of PH. Current guidelines for the management of valvular heart disease [27] do not advise routine right heart catheterization prior to AVR for AS. Thus, right heart catheterization was not performed consistently enough to include these data in the analysis. These data could also have given a clearer perspective concerning left ventricular filling pressures. To compensate for this limitation, we analyzed left atrial size. Left atrial size has previously been described as the “barometer of the left ventricle”, since left ventricular end-diastolic pressure is the main determinant of left atrial size [28, 29]. Left atria were significantly larger in patients with a relevant TR. Furthermore left atrial size was significantly correlated with peak TR velocity.

However, while the estimation of pulmonary artery pressures by echo may be imprecise, the detection of more than mild TR should be more reliable. As TR indicates not only a rise of the pulmonary artery pressure but also a change of RV geometry, it may be a sign of incipient RV failure.

Whether patients with significant TR may also benefit from a surgical correction of TR at the time of AVR is unclear, longitudinal data will be necessary to clarify this question.

### Limitations

Our data have been collected in a single center, therefore a center-specific bias cannot be excluded. On the other hand, major advantages of limiting data collection to a single center are: inclusion of a homogenous patient population, adherence to a constant clinical routine, constant quality of echocardiographic work-up, and constant surgical quality over a time-period of 11 years.

In patients with severe AS, left ventricular longitudinal function is often impaired despite normal ejection fraction and this dysfunction has prognostic value [30]. The present study is limited by the lack of information on left ventricular longitudinal function.

Quantification of TR was performed by both qualitative and semi-quantitative measurements. Nevertheless, exact quantification may be demanding. To account for potential inaccuracy TR severity was divided into significant and non-significant TR for further analysis.

This approach has previously been deemed reasonable [1, 3, 5, 9]. However, patients summarized in the group of significant TR were heterogeneous with a spectrum of TR severities. In addition, a significant proportion of patients in the TR group had already developed RV dysfunction, which also causes further sample variability.

Furthermore, only 0.7% of patients underwent concomitant tricuspid valve surgery. In the absence of a clear recommendation concerning tricuspid valve repair in patients undergoing aortic valve surgery in the 2006 AHA guidelines [31], tricuspid valve repair in our patients was performed according to the surgeon´s discretion.

### Conclusions

Significant TR is associated with outcome in patients undergoing AVR for severe AS, in addition to RVD, age, and CABG. This finding underlines the importance of a thorough echocardiographic evaluation with particular consideration of the right heart in these patients.

## Supporting Information

S1 STROBE Checklist This manuscript is in compliance with the STROBE statement. All applicable points have been addressed.
(DOC)
