# Supplementary material for: Prognostic Impact of Tricuspid Regurgitation in Patients Undergoing Aortic Valve Surgery for Aortic Stenosis
Source: PLoS One. 2015 Aug 20;10(8):e0136024. doi: 10.1371/journal.pone.0136024 (PMC4546400; doi:10.1371/journal.pone.0136024)
Supplement: S1 STROBE Checklist — All applicable points have been addressed. (DOC) [file pone.0136024.s001.doc]

STROBE Statement—Checklist of items that should be included in reports of ***cohort studies***

|  | Item No | Recommendation |
| --- | --- | --- |
| **Title and abstract** | 1 | (*a*) Indicate the study’s design with a commonly used term in the title or the abstract: *Page 2* |
|  |  | (*b*) Provide in the abstract an informative and balanced summary of what was done and what was found: *Page 2* |
| Introduction | | |
| Background/rationale | 2 | Explain the scientific background and rationale for the investigation being reported: *Page 5* |
| Objectives | 3 | State specific objectives, including any prespecified hypotheses: *Page 5* |
| Methods | | |
| Study design | 4 | Present key elements of study design early in the paper: *Page 6* |
| Setting | 5 | Describe the setting, locations, and relevant dates, including periods of recruitment, exposure, follow-up, and data collection: *Page 6* |
| Participants | 6 | (*a*) Give the eligibility criteria, and the sources and methods of selection of participants. Describe methods of follow-up: *Page 6* |
|  |  | (*b*) For matched studies, give matching criteria and number of exposed and unexposed: N/A |
| Variables | 7 | Clearly define all outcomes, exposures, predictors, potential confounders, and effect modifiers. Give diagnostic criteria, if applicable: *Pages 6,7* |
| Data sources/ measurement | 8* | For each variable of interest, give sources of data and details of methods of assessment (measurement). Describe comparability of assessment methods if there is more than one group: *Page 7* |
| Bias | 9 | Describe any efforts to address potential sources of bias: *Page 8* |
| Study size | 10 | Explain how the study size was arrived at: N/A |
| Quantitative variables | 11 | Explain how quantitative variables were handled in the analyses. If applicable, describe which groupings were chosen and why: *Page 8* |
| Statistical methods | 12 | (*a*) Describe all statistical methods, including those used to control for confounding: *Page 8* |
|  |  | (*b*) Describe any methods used to examine subgroups and interactions: *Page 8* |
|  |  | (*c*) Explain how missing data were addressed: N/A |
|  |  | (*d*) If applicable, explain how loss to follow-up was addressed: *Page 11* |
|  |  | (*e*) Describe any sensitivity analyses: N/A |
| Results | | |
| Participants | 13* | (a) Report numbers of individuals at each stage of study—eg numbers potentially eligible, examined for eligibility, confirmed eligible, included in the study, completing follow-up, and analysed: *Pages 10,11* |
|  |  | (b) Give reasons for non-participation at each stage: N/A |
|  |  | (c) Consider use of a flow diagram: N/A |
| Descriptive data | 14* | (a) Give characteristics of study participants (eg demographic, clinical, social) and information on exposures and potential confounders: *Pages10,11* |
|  |  | (b) Indicate number of participants with missing data for each variable of interest: N/S |
|  |  | (c) Summarise follow-up time (eg, average and total amount): *Pages10,11* |
| Outcome data | 15* | Report numbers of outcome events or summary measures over time: *Pages11, 12* |
| Main results | 16 | (*a*) Give unadjusted estimates and, if applicable, confounder-adjusted estimates and their precision (eg, 95% confidence interval). Make clear which confounders were adjusted for and why they were included: *Page 27* |
|  |  | (*b*) Report category boundaries when continuous variables were categorized: N/A |
|  |  | (*c*) If relevant, consider translating estimates of relative risk into absolute risk for a meaningful time period: N/A |
| Other analyses | 17 | Report other analyses done—eg analyses of subgroups and interactions, and sensitivity analyses: N/A |
| Discussion | | |
| Key results | 18 | Summarise key results with reference to study objectives: *Page 13* |
| Limitations | 19 | Discuss limitations of the study, taking into account sources of potential bias or imprecision. Discuss both direction and magnitude of any potential bias: *Page 15* |
| Interpretation | 20 | Give a cautious overall interpretation of results considering objectives, limitations, multiplicity of analyses, results from similar studies, and other relevant evidence: *Pages13, 14* |
| Generalisability | 21 | Discuss the generalisability (external validity) of the study results: *Page 15* |
| Other information | | |
| Funding | 22 | Give the source of funding and the role of the funders for the present study and, if applicable, for the original study on which the present article is based: *Page 17* |

*Give information separately for exposed and unexposed groups.
